# Identification of Berenil Target Sites in Plasmid pBR322

**DOI:** 10.19070/2332-2756-170004

**Published:** 2017-05-11

**Authors:** MS Valenzuela, N Green, S Liu

**Affiliations:** Department of Biochemistry and Cancer Biology, School of Medicine, Meharry Medical College, 1005 DB Todd Jr. BIvd, Nashville, TN, USA

## Abstract

Berenil, a minor groove DNA binding molecule, has been extensively used in veterinary medicine. Modeling studies have suggested that berenil binds to A/T rich regions on the DNA and the product of this interaction causes the formation of crosslinks between opposite DNA strands. These crosslinks could potentially inhibit fundamental biological processes including transcription and DNA replication. We had previously used the pBR322 genome as a model system to investigate the role of A/T sequences on berenil activity. We reported that the insertion of poly(dA)poly(dT) sequences into the pBR322 genome causes replication inhibition of the recombinant plasmids when cultures were exposed to berenil. However, we noticed that even in the absence of these sequences the parental plasmid replication was also inhibited, albeit less than the recombinants. This observation led us to the present study were we attempted to identify the location of natural berenil target sites in the pBR322 genome. Through a combination of deletion analysis, recombinant DNA and a replication assay we uncovered a 378 bp DNA fragment that has all the hallmarks of a berenil target site. A recombinant plasmid lacking this region is more refractive to the drug than the parental plasmid, and another variant containing and extra copy of this region increases the susceptibility of the plasmid towards berenil. The 378 bp region is about 60% A/T rich and contains about 21 potential berenil binding sites.

## Introduction

Berenil {4-4′-(1-triazene-1,3-dyil)bis(benzamidine)}, an aromatic diamidine [1], displays a trypanocidal, and bactericidal activity, and has been used in veterinary medicine for the treatment of trypanosomiasis [2–4]. Studies on the binding of this compound to oligomeric polynucleotides have indicated that it belongs to a class of minor-groove DNA binding drugs with a preference for A-T rich sequences [5–8]. Modeling studies have suggested that berenil binding is through its participation in the formation of hydrogen bond-bridges between opposite A/T nucleotides in the DNA double helix) [9]. These additional hydrogen bonds, could potentially act as inhibitors of processes that fundamentally depend on DNA melting, including transcription and DNA replication. The pioneering *in vivo* studies of Brack et al., [10] pointed to a direct effect of berenil on DNA replication by the observation of a thousand–fold increase in the accumulation of mitochondrial minicircle DNA replicative intermediates when a T.cruzi culture had been exposed to the drug. The high A-T composition of minicircle DNA, a component of mitochondrial kinetoplast DNA [11] offered an explanation for the specific effect of the drug on these DNA species. Later studies however indicated that topoisomerases might also be the targets for the drug [12–16] which could also lead to the observed accumulation of minicircle replicative intermediates. In an effort to discern the DNA binding effect of berenil we constructed recombinant plasmids containing poly(dA)poly(dT) sequences and studied the effect of the drug on the replication of these recombinant plasmids vis a vis the parental genome. We demonstrated that the presence and position of a poly(dA)poly(dT) homopolymer on the pBR322 plasmid genome, determine the degree of arrest of DNA replication by the drug [17–19]. We however observed that even in the absence of the poly(dA)poly(dT) sequence, the parental plasmid itself is sensitive to the drug, thus natural berenil target sites must be present in the pBR322 genome [17].

In this study we attempted to gain a better understanding about the mechanism of action of berenil on natural genomes by identifying the location of its target sites in the pBR3222 genome. Using a combination of deletion analysis, recombinant DNA, analysis of newly synthesized DNA, and real-time PCR techniques, we have uncovered a 378 bp fragment that appears to be responsible for berenil sensitivity. A deletion mutant lacking most of this fragment showed to be more resistant to berenil than the parental plasmid. Conversely, addition of the 378 bp fragment to pBR322 rendered recombinant plasmid more sensitivity towards the drug compared to the parental plasmid. Analysis of newly synthesized plasmid DNA by Cs_2_SO_4_ equilibrium density centrifugation in the presence and absence of berenil indicated that the presence of the target region causes a significant decrease in the replication of plasmid DNA. Finally, we have also observed that in the presence of berenil, PCR amplification of this fragment is considerably reduced.

## Materials and Methods

### Bacterial Strains and Plasmid Preparation

*E.coli* strain HBT [20], a thymine-requiring derivative of HB101 [21] was used for transformations and propagation of recombinant plasmids. Large-scale isolation and purification of plasmid DNA was performed by a modification of the cleared lysate method [22] followed by centrifugation to equilibrium in a Cesium Chloride-Ethidium Bromide density gradient. Small aliquots of plasmid DNA were isolated by a modification [23] of the boiling method [24]. Plasmid DNA was then purified using a GeneClean kit (Bio101, La Jolla CA) following the manufacturer’s instructions.

### Plasmids used in this Study

Plasmid pBR322 was purchased from Bethesda Research Laboratories (Bethesda, MD). Plasmid pBR322DraIDelta containing a 711 bp deletion was constructed by digesting pBR322 with restriction enzyme DraI coupled with self-ligation of the major DNA fragment in the presence of ligase. HBT was then transformed with the ligation product and colonies that were resistant to tetracycline but sensitive to ampicillin were chosen. The presence of the deletion was further confirmed by analyzing the plasmid after digestion with DraI restriction enzyme. Plasmid pBR322Dra was constructed by inserting a 692 bp fragment produced by the digestion of pBR322 with DraI, into the PvuII site of plasmid pBR322 following standard protocol. For the construction of plasmid pBR322Bgl a 378 bp fragment comprising (nucleotides 3359-2981) from the pBR322 DNA sequence, was amplified by PCR using forward (aagagatctatttggtatctgcgctctgc) and reverse (gggagatctggcaactatggatgaacgaaa) primers. The amplified product was then inserted into the plasmid pBR322 Dra previously digested with BamH1, following standard practice.

### Berenil Assays

Berenil (diminazene aceturate) was purchased from Sigma (St. Louis, MO). *E.coli* HBT cells containing plasmids were grown in LB-ampicillin medium. Starting at an OD_600_ of about 0.05, berenil was added to the cultures, aliquots taken at one hour intervals and dilutions plated in LB-Ampicillin plates in triplicate, to monitor the effect of the drug on bacterial growth. To measure the effect of berenil concentration on bacterial growth cultures containing different concentrations of berenil were stopped at about 5 hrs. after incubation and dilutions of the cultures at each concentration were plated in triplicate.

### Determination of Plasmid Copy Number

A quantitative real time PCR assay was used as previously described [25] to determine the number of plasmid DNA molecules present in a bacterial culture. After plasmid isolation, a dilution of the DNA sample previously digested to completion with EcoRI was used as a template on a standard protocol using a 50 ul SybrGreen PCR Master Mix (Applied Biosystems) containing 6.25pmol of each, forward primer (pBR1732: ggcattgaccctga), and reverse primer (pBR1882: tgggggtaatgataccgat) and run on a CFX96 Real Time PCR System (BioRad). The PCR program consisted of an initial denaturation at 94°C for 30 sec followed by 35 cycles of 94°C 30 sec; 55°C 45 sec; and 68°C 45 sec. At the end of the run initial copy number values were calculate. Alternatively amplified DNAs at the end of the run were analyzed on a 2% agarose gel following standard methodology.

### Analysis of Plasmid DNA Replication by C_2_SO_4_ Equilibrium Density Gradients

Overnight bacterial cultures were diluted 100-fold into 100 ml of fresh LB medium containing thymidine and ampicillin and grown at 37°C up to OD_600_= 0.6. Chloramphenicol (170ug/ml) was added to the cultures followed by incubation at 37°C for 30 min. Cultures were divided into two flasks. To one of the flasks berenil (20ug/ml) was added and incubation continued in both flasks for an additional 30°min. Cells were pelleted by centrifugation, washed twice with LB-medium and resuspended in 5 ml LB medium containing chloramphenicol (170ug/ml) and bromodeoxyuridine (BUdR, at 5ug/ml). One of the flasks contained in addition berenil (20ug/ml). After incubation for 10 min at 37°C, cultures were added to frozen sodium azide (0.05 M in TE buffer) and pelleted by centrifugation at 4°C. Pellets were resuspended in 5ml TE buffer and 30 ul of lysozyme (10 ug/ml in 10 mM Tris, pH 8) was added. Cells were frozen and thawed three times in liquid nitrogen and under cold running water respectively, and placed on ice. 6 ul of Triton X-100 was added, mixed gently, and the resulting lysate centrifuged at 30K rpm for 30 min at 4°C to pellet the bacterial DNA. The supernatant was treated with 300 ul Pronase E (20 mg/ml) and incubated at 37°C for 60 min. The resulting suspension was brought to a refractive index of 1.368 with solid C_2_SO_4_. The suspension was then centrifuged at 36K rpm for 44 hr. at 12°C in a Beckman VTi65 rotor. At the end of the run fractions containing about 250 ul were collected from top to bottom using an ISCO fraction collector. The refractive index of each fraction of every other fraction was then measured, refractive index values were plotted and a regression line was calculated from the curve. Refractive index values corresponding to the regression line for each fraction were then calculated in order to cancel out differences among samples. To determine the relative amount of plasmid DNA in each fraction. 200 ul from each fraction was made 0.4 M NaOH and dot-blotted unto a Schleicher & Schuell filtration manifold (Schleicher & Schuell, Keene, NH). Membranes were then hybridized to a 32P-labeled pBR322 DNA probe (prepared by a standard methodology [26]) following standard protocol. Radioactivity in each of the dot blots were calculated using a densitometer scanner. Relative intensity values were then calculated and these numbers plotted against the fraction number corresponding to each dot. An analysis of each of the fractions from the gradient using quantitative real time PCR to determine plasmid copy number, yielded similar results.

### PCR Amplification of Target Region in the Presence of Berenil

50ul PCR system containing 1× Taq Master Mix (New England BioLabs) with additional 1uM MgCl_2_, 6.25pmol of both forward (aagagatctatttggtatctgcgctctgc) and reverse primer (gggagatctggcaactatggatgaacgaaa), 5pg of purified 387 bp fragment (nucleotides 3359-2981 in pBR322), and berenil at final concentrations of 0,1, 10, and 100uM, were used in a regular PCR reaction. The PCR program consisted on an initial denaturation at 94°C for 30 seconds followed by 30 cycles of 94°C 30 seconds 55°C 45 seconds and 68°C 45 seconds. Tubes were taken out after 24^th^, 27^th^, and 30^th^ cycle and then run on a 2% agarose gel.

## Results

### Identification of Berenil Target Regions in pBR322

**Figure 1:**
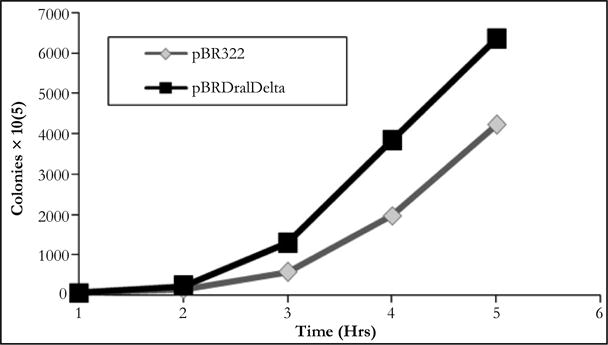
Growth of *E.coli* HBT cells containing plasmid pBR322 or its derqivative pBRDraIDelta in the presence of 20 ug/ml berenil.

**Figure 2:**
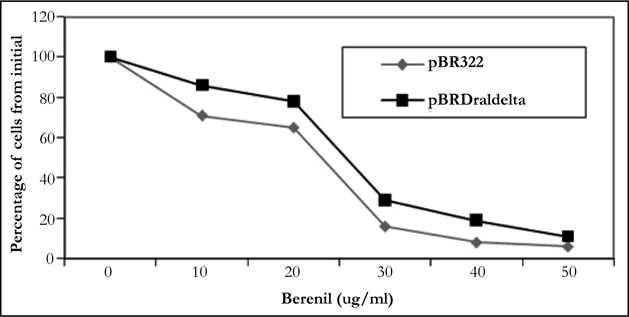
Recovery of HBTpBR322) and HBT(pBRDraIDelta) colonies on plates containing indicated concentrations of berenil.

As indicated earlier, it had been demonstrated that berenil can potentially bind DNA regions containing A-T rich tracks. In agreement with this, previous work in our laboratory had indicated that the insertion of poly(dA)poly(dT) sequences in pBR322 renders the resulting plasmids more sensitivity to the drug compared to the parental plasmid. This sensitivity was demonstrated by the reduced plasmid yield obtained with both parental and recombinant plasmids, which was shown to be in great in part derived from an inhibition of plasmid DNA replication by the drug [17–19]. To identify the natural berenil target sites in pBR322 we decided to focus on a 1.6 kb DNA fragment from a PBR322 related plasmid which in an unrelated study had been shown to stain preferentially with berenil [27]. On the pBR322 genome, this fragment mapped to a region covering the ampicillin resistance gene. Inspection of the DNA sequence suggested a possible explanation for the binding of berenil to this region since it contained several A/T rich tracts. The most predominant of these tracts was located around a region containing three DraI sites (nucleotides 3230-3941). We hypothesized that if these tracts were relevant to berenil binding, their removal should make the resultant plasmid more refractive to the drug. To test this hypothesis we removed the 692 bp flanked by these restriction sites followed by self-ligation of the reminder plasmid DNA to yield plasmid pBR Delta. The sensitivity to berenil of the resulting plasmid was first probed by comparing the growth rate of *E.coli* HBT containing either the deletion plasmid mutant (pBR Delta) or the parental plasmid (pBR322) in LB media supplemented with tetracycline, in the presence of 20 ug/ml berenil. As shown in Figure 1, the growth inhibition induced by berenil in pBR322 containing cells was significantly attenuated in the deletion mutant. We also compared the colony formation capacity of both strains on agar plates containing different berenil concentrations. As shown in Figure 2, the recovery of cells in the deletion mutant is consistently higher than in the parental plasmid. We therefore concluded that the DNA region contained in the deleted fragment must harbor target sites for berenil.

### Effect of Berenil on Recombinant Plasmids Containing Target Sites

**Figure 3:**
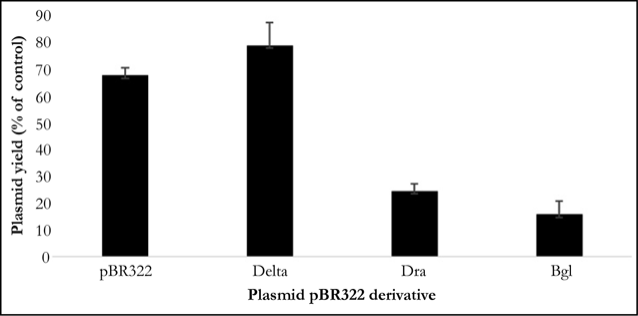
Plasmid yield obtained from cultures containing HBT(pBR322), HBT(pBRDraIDelta), HBT(pBRDraI), and HBT(pBRBgl), indicated in the figure as pBR322, Delta, Dra and Bgl, respectively, the presence of 20 ug/ml berenil. The amount of plasmid yield recovered was plotted relative to that found in the culture grown in the absence of berenil. The error bars indicate the standard deviations obtained with triplicate samples.

To determine that the observed effect of bacterial growth was due to a direct effect of berenil on the plasmids, we used real time PCR to compared the amount of plasmid DNA recovered from bacterial cultures that had been previously treated with chloramphenicol followed by addition of berenil at 20 ug/ml *vis a vis* similar cultures that had not been exposed to berenil. As shown in Figure 3 (for simplicity, in Figure 3 the pBR322-derived recombinants are labeled as Delta, Dra and Bgl, respectively) the amount of plasmid DNA recovered from pBR Delta is significantly higher than the parental pBR322 plasmid, suggesting that the loss of the 692 bp fragment made the resulting plasmid more resistant to the inhibitory effect of berenil. We then introduced the 692 bp region at the EcoRV site in pBR322, to generate the plasmid pBR Dra harboring two copies of the 692 bp region, which we assumed would render the resulting plasmid more sensitivity to the drug. Figure 3 shows than in contrast to pBRDelta, pBRDra yielded a four-fold less plasmid DNA in presence of berenil. These results indicated that the 692 bp region constitutes a *bonna fide* berenil target site. Upon inspection of the nucleotide sequence around the DraI sites in pBR322, we noticed that several tracts of putative berenil binding sites surrounding the two DraI sites had not been included in the pBRDra construct. We amplified this 378 bp region (nucleotides 2981-3359 in the pBR322 genome) and flanked them with terminal BglII restriction enzyme sites in order to insert the fragment at the single BamHI site present in pBR322 to generate the plasmid pBRBgl. In the presence of berenil, HBT cells harboring plasmid pBRBgl yielded the lowest number of plasmid copies compared to pBRDra (Figure 3), indicating that the duplication of this region confers the highest sensitivity to berenil.

### Inhibition of DNA Replication by Berenil in Recombinant Plasmids Containing Target Sites

**Figure 4:**
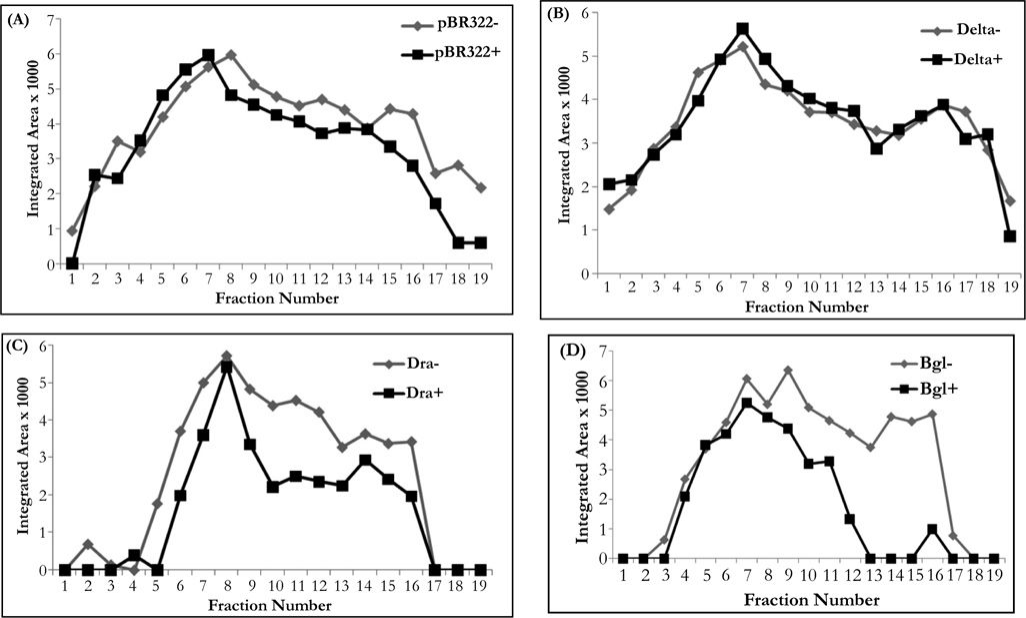
Dot blot analysis of BUdR incorporation into plasmid DNA in the presence and absence of 20 ug/ml berenil. BUdR incorporation into plasmid DNA was followed in bacterial cells previously treated with chloramphenicol. Plasmid preparations from these cultures were then fractionated from top to bottom after a Cs_2_SO_4_ equilibrium density centrifugation. To compare the incorporation in cultures exposed (+) or not (−) to the drug, the relative intensity of plasmid DNA present in each fraction which hybridized to a radioactive probe (integrated area) was plotted against the fraction number. The peak around fraction 7–8 corresponded to the density of the parental DNA, whereas the signal above fraction 8 correspond to fractions with higher densities containing newly synthesized DNA. Plasmid DNAs were isolated from HBT cultures containing (A) pBR322; (B) pBRDraIDelta (labeled Delta in the figure); (C) pBRDra (labeled Dra in figure); and (D) pBRBgl (labeled Bgl in the figure). The + and − signs following the plasmid labels indicate plasmid DNA recovered from cultures that were or not exposed to berenil.

Previous studies on the sensitivity of pBR322 derivatives containing additional poly(dA)poly(dT) sequences had shown that the low plasmid yield of these plasmids when cultures were exposed to berenil could be explained by an impairment in plasmid DNA replication. This conclusion was reached by measuring the incorporation of BUdR into DNA, in the presence and absence of berenil, followed by a C_2_SO_4_ equilibrium density centrifugation to distinguish newly synthesized from parental DNA [17–19]. This same approach was applied to the pBR322 derivatives constructed in this study. To this end the effect of berenil on the replication of plasmids pBR322, pBRdelta, pBR Dra and pBRBgl was analyzed by comparing the incorporation of BUdR in newly synthesized plasmid DNA produced in both the absence or presence of berenil. To ensure that the incorporation of BUdR occurred primarily on plasmid DNA, the cultures were previously treated with chloramphenicol to arrest bacterial DNA replication (see Materials and Methods section). In all runs the linearity of the gradient in the matched samples (with or without berenil) was determined by measuring the refractive index in each other fraction. The regression line obtained with each matched sample was equivalent, indicating that the density of each fraction was similar in the matched samples. More importantly, in all samples the density profile along the gradient gave a similar regression line. Based on these results we inferred that fractions 7–8 in all gradients contained the parental DNA, whereas upper fractions contained newly synthesized DNA. Figure 4 shows the profiles of matched samples for each of the pBR322 derivatives (for simplicity in Figure 4 the pBR322 derivatives are labeled as Delta, Dra, and Bgl, respectively. The signs − and + following the plasmid label indicate the samples without and with berenil, respectively). Our results are consistent with the plasmid copy number results obtained for each of the plasmids when the cultures were exposed to berenil (see Figure 3 above). Thus, while in the parental pBR322 plasmid we observed a reduction in the amount of newly synthesized DNA in the culture containing berenil (Figure. 4A, pBR322+), this reduction is ameliorated in the pBR322Delta variant where a portion of the target DNA has been deleted (Figure 4B; Delta+). In contrast, in pBR322Dra in which a duplicated portion of the target region has been inserted, the reduction of newly synthesized DNA is greater than in pBR322 (Figure 4C; Dra+); and this effect is even more pronounced in plasmid pBR322Bgl which contains an extra full complement of the target region (Figure. 4D; Bgl+). It is therefore apparent that the reduction of plasmid yield in the constructs containing the berenil target site is due to an interference of the drug with some aspect of the DNA replication process *per se*.

Plasmid preparations from these cultures were then fractionated from top to bottom after a Cs_2_SO_4_ equilibrium density centrifugation. To compare the incorporation in cultures exposed (+) or not (−) to the drug the relative intensity of plasmid DNA present in each fraction which hybridized to a radioactive probe (integrated area) was plotted against the fraction number. The peak around fraction 7–8 corresponded to the density of the parental DNA, whereas the signal above fraction 8 correspond to fractions with higher densities containing newly synthesized DNA. Plasmid DNAs were isolated from HBT cultures containing (A) pBR322; (B) pBRDraIDelta (labeled Delta in the figure); (C) pBRDra (labeled Dra in figure); and (D) pBRBgl (labeled Bgl in the figure). The + and − signs following the plasmid labels indicate plasmid DNA recovered from cultures that were or not exposed to berenil.

### Interference of Berenil with the Amplification of Target DNA

**Figure 5:**
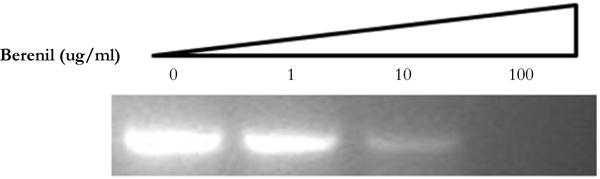
PCR amplification product obtained with template 378 bp DNA fragment in the presence of 0, I, 10, and 100 ug/ml berenil. The single product obtained after running on a 2% agarose gels was visualized with Ethidium Bromide, and its sized confirmed using DNA size markers (not shown). The DNA products shown were obtained after a 30 cycle PCR amplification reaction.

In an unrelated study we had observed that DNA binding drugs interfere with the PCR-mediated amplification of DNA fragments containing their drug-specific sequences (Valenzuela and Liu, unpublished). We therefore decided to test if the same would hold true for a berenil target. To this end, the 378 bp fragment which had shown to be an important berenil target was PCR amplified in the presence of varying concentrations of berenil. As shown in Figure 5, there is a dramatic reduction in the amount of amplified DNA recovered in the reactions containing berenil. About a twofold reduction was obtained at 1ug/ml berenil; ten-fold at 10 ug/ml; and at 100 ug/ml no amplified DNA was recovered. Also this amplification was not altered by berenil if a G-C rich fragment was used as template (data not shown). This result indicates that berenil can directly affect the activity of the DNA polymerase, possibly by reducing the efficiency of the opening of the DNA strands, a requirement for the amplification reaction.

## Discussion

Berenil, a trypanocidal agent, has been shown to interact with DNA at regions where at least four A/T residues at present [8]. Modeling studies with synthetic polymers has suggested that the binding of the drug at these sites has the potential of generating cross-links between opposite strands of a double stranded molecule. In this context, the trypanocidal activity of berenil had been ascribed to its efficient binding to minicircle DNA (the major component of kinetoplast DNA (kDNA)), which is about 60% rich in A/T sequences. *In vivo* studies by Brack et al., [10] showed that the drug inhibits the replication of minicircle kDNA, causing the accumulation of replicative intermediates. Other studies have suggested however that the impairment of DNA replication could also be explained by the inhibition of topoisomerases by berenil [14–16].

In order to ascertain that A/T rich DNA sequences are *in vivo* berenil target sites, we studied the effect of the drug on the replication of recombinant pBR322 derived plasmids containing poly(dA)poly(dT) homopolymers, by both measuring plasmid yield and a density shift analysis of newly replicating plasmid DNA, when bacterial cultures containing these plasmids were exposed to the drug. We found that in comparison to the parental plasmid pBR322, the recombinant plasmids exhibited both a significant reduction in yield as well as in their replication potential when exposed to berenil. However we noticed that the parental plasmid, pBR322, was also affected (albeit to a lesser degree) indicating that it harbored berenil target sites [17–19].

**Figure 6:**
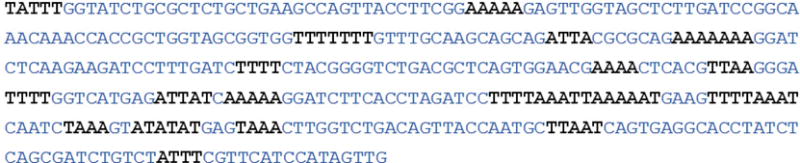
Nucleotide sequence of the putative 378 bp berenil target region from pBR322. Runs of consecutive A/T residues (n ≥ 4) are highlighted in bold. These correspond to potential berenil binding sites.

In this study we have attempted to localize putative berenil target sites in the pBR322 genome. We first focused our attention on a 1.6 kb fragment that in an unrelated study had been found to preferentially stain with berenil [27]. This fragment mapped to a region that included the ampicillin resistant (bla) gene. Upon inspection of the DNA sequence around this region, we noticed several tracts of A/T rich regions within the three Dra I restriction enzyme sites (coordinates 3230-3941) present in the plasmid. We found that upon deletion of this DraI surrounding region, to yield the plasmid pBRDelta, the bacteria containing the deletion variant was more resistance to the drug than the parental plasmid, indicating that a putative berenil target site was present in this region. Upon further analysis of this region, our studies narrowed down on a 378 bp fragment (coordinates 2981-3359) which had all the hallmarks of a putative berenil target site. First, in presence of the drug, deletion of part of this region improved the plasmid yield compared to the parental plasmid; conversely insertion of this region into the pBR322 genome decrease considerably the plasmid yield of the resultant recombinant; Second, the extra presence of this region in the parental plasmid caused a significant reduction in the production of newly synthesized DNA, similar to the results obtained with pBR322 derivatives containing poly(dA) poly(dT) homopolymers [17]; Third, the 378 region is about 60% rich in A/T sequences, which is similar to that found to minicircle kDNA, the best known DNA target for berenil. Modeling studies have suggested that about a molecule of berenil could interact with four A/T nucleotides at the minor groove of DNA [6–8]. Inspection of the sequence of the 378 bp fragment, shown in Figure 6, suggests that it contains 21 potential berenil binding sites (the sites are indicated in bold letters) that is, this fragment on average harbors more than one berenil binding site per each 20 base pairs. Lastly, the specific inhibition of PCR amplification of the 378 fragment in the presence of berenil provides a direct evidence of the direct interaction of this fragment with the drug and suggests that the mechanism by which berenil interferes with DNA replication may not depend solely on its anti-topoisomerase activity.

In our previous study where poly(dA)poly(dT) homopolymers had been introduced into the pBR322 genome, we observed that the location of the insertion site determined the inhibitory effect of berenil on the replication of the plasmid. Thus plasmid pKH47 containing 100 bp of the homopolymer at the PvuII site in pBR322 showed a much higher sensitivity to the drug than plasmid pVL26, containing 240 bp of the homopolymer at the EcoRV site in pBR322 [17]. We reasoned that since replication in pBR322 occurs in a unidirectional fashion starting at a unique origin located 500 bp upstream from the PvuII site, early replicative forks traveling toward in an anticlockwise fashion would encounter the homopolymer present in pKH47, whereas late replicating forks would encounter this region in plasmid pVL26. Thus in pKH47 forks would be arrested very early on in the replication process thus explaining the major sensitivity of this plasmid to berenil as detected in our replication assay. In this context, the pattern of DNA replication inhibition observed with pBRBgl (where the 378 bp had been positioned 188 bp downstream from the EcoRV site) is very similar to that obtained with plasmid pVL26 provides further evidence that the 378 bp fragment is indeed a target site for the drug.

Finally, we should also point out that in our previous studies with the poly(dA)poly(dT) homopolymers we reported that these sequences were not very stable such that recurrent deletions in both plasmids pKH47 and pVL26 were observed [18, 28]. In contrast, we have not observed such instability in the recombinants containing the 378 bp fragment. Therefore, in the construction of genomes harboring target sequences, natural targets such as the 378 bp region should be the sequences of choice.

## Conclusions

In this study we have uncovered a 378 bp DNA sequence present in the pBR322 genome which has the properties of a putative berenil target site. The DNA sequence of this region shows that is 60% A/T rich and that it contains about 21 potential berenil binding sites. This work should pave the way for the development and/or identification of ‘poison’ DNA sequences which upon insertion into a desired target genome will make it susceptible to a drug that recognizes the ‘poison’ DNA sequence.
